# Amalgam Pills

**Published:** 1899-10

**Authors:** W. Andre Campbell

**Affiliations:** Brooklyn, N. Y.


ARTICLE IV.

### AMALGAM PILLS.

W. ANDRE CAMPBELL, M. D. S., BROOKLYN, N. Y.

The component parts of amalgam pills in olden times, that is, my early dental days, consisted of two parts of granulated silver to one part of pure tin. And how surprising to find how varied the product might be from this simple formula. No less than three varieties are produced in the fusion alone, for if the silver is overheated, when the tin is added, a portion of it (the tin) is lost in the fuse; if the silver is overheated, an imperfect amalgamation is the result; but with a proper temperature, then the perfect product, and that is easily spoiled or changed, for if the melted mass is poured into an overheated or underheated ingot (the specific gravity of the two metals being so different), this will also cause an imperfect product. So it is difficult to get a uniform composition, and when obtained, the fillings or shavings can again be robbed of some of their good qualities by an excess or an insufficient quantity of quicksilver. Therefore, is it possible to obtain a positive and perfect amalgam, even from any of the up-to-date formulas ?

Possibly Dr. Black *may* know (manufacturers of course *do* know), and possibly the present scientific methods have overcome these difficulties. My experience and observation, however, do not warrant the assumption.

There is no doubt that there are good productions, at times, and good manipulation also from any of the formulas, for I have seen a filling which stood the test of use for thirty years, and was then in perfect condition. This, of course, must have been made from the original simple formula.

Mercurial poisoning from amalgam pills, as has been claimed, I decline to believe. The original formula required a large proportion of quicksilver to make it plastic, and always mixed in the palm of the hand, which I have done for the past thirty-five years, and have noticed no bad results to patient or myself.

In my early days of dentistry, I had an opportunity to fill for a brother dentist a superior third molar. I was about to place the pill (which was as large as a good sized pea) in position (they were always made in that form in those days and put in the cavity whole, and in a very soft mass), when I had the pleasure of seeing it fall off my fingers (it was usually carried by the finger at that time) and quietly and gently roll down his esophagus. There was no effect, either bad or good, from the voluntary dose. He is still living.

I am inclined to believe Dr, Tuthill's (physician) experience with this kind of "pill," and its bad results—mercurial poisoning—which he claims were in his teeth thirty-eight years, is a case of imperfect diagnosis. He says he had *them* removed. What could he have had removed? If the system had already absorbed *them*, and if the system was already surcharged with mercury, how could he find relief by adding more, as he says he did, by using bichlorid of mercury as a dressing? However, it may be an idiosyncrasy of his; and we will be charitable.—*Items of Interest.*

